# Chromosome conformation capture approaches to investigate 3D genome architecture in Ankylosing Spondylitis

**DOI:** 10.3389/fgene.2023.1129207

**Published:** 2023-01-25

**Authors:** Connor Davidson, B. Paul Wordsworth, Carla J. Cohen, Julian C. Knight, Matteo Vecellio

**Affiliations:** ^1^ Wellcome Centre of Human Genetics, University of Oxford, Oxford, United Kingdom; ^2^ Nuffield Department of Orthopaedics, Rheumatology and Musculoskeletal Sciences, Botnar Research Centre, University of Oxford, Oxford, United Kingdom; ^3^ MRC WIMM Centre for Computational Biology, MRC Weatherall Institute for Molecular Medicine, University of Oxford, Oxford, United Kingdom; ^4^ Centro Ricerche Fondazione Italiana Ricerca Sull’Artrite (FIRA), Fondazione Pisana x la Scienza ONLUS, San Giuliano Terme, Italy

**Keywords:** genomics, three dimensional genome, ankylosing spondylitis, chromosome conformation capture (3C), topologically associated domain (TAD), rheumatic and musculoskeletal disease

## Abstract

Ankylosing Spondylitis (AS) is a chronic inflammatory arthritis of the spine exhibiting a strong genetic background. The mechanistic and functional understanding of the AS-associated genomic loci, identified with Genome Wide Association Studies (GWAS), remains challenging. Chromosome conformation capture (3C) and derivatives are recent techniques which are of great help in elucidating the spatial genome organization and of enormous support in uncover a mechanistic explanation for disease-associated genetic variants. The perturbation of three-dimensional (3D) genome hierarchy may lead to a plethora of human diseases, including rheumatological disorders. Here we illustrate the latest approaches and related findings on the field of genome organization, highlighting how the instability of 3D genome conformation may be among the causes of rheumatological disease phenotypes. We suggest a new perspective on the inclusive potential of a 3C approach to inform GWAS results in rheumatic diseases. 3D genome organization may ultimately lead to a more precise and comprehensive functional interpretation of AS association, which is the starting point for emerging and more specific therapies.

## Introduction

The combination of environmental and genetic factors may lead to the development of complex diseases (Hunter, 2005). Ankylosing Spondylitis (AS) is a common form of arthritis primarily affecting the spine, characterised by inflammation at the entheses (Bridgewood et al., 2019) and sacroiliac joints (Brewerton et al., 1973). AS is a highly heritable disease with more than 100 genomic loci found implicated in increasing the risk (International Genetics of Ankylosing Spondylitis Consortium et al., 2013; Ellinghaus et al., 2016). Genome wide association studies (GWAS) have been very successful in polygenic disease as they identified thousands of common genetic variants or single nucleotide polymorphisms (SNPs), which can have a phenotypical individual effect (Huo et al., 2019; Crouch and Bodmer, 2020). The identification of a causal variant from GWAS data may help our understanding of complex traits biology, suggesting new target genes and methods of controlling them. Unfortunately, disease-associated loci often contain multiple genes making the scenario extremely challenging; genetic variants in proximal vicinity tend to be inherited together, in a phenomenon called linkage disequilibrium (LD), making difficult to identify the causal variant underpinning the association (Cano-Gamez and Trynka, 2020). In recent years, large-scale epigenomic projects have mapped hundreds of thousands of potential regulatory sites in the human genome, but only a small proportion of these elements are proximal to transcription start sites (Bagchi and Iyer, 2016). In AS, we and others were able to successfully identify the causal functional SNPs at the Interleukin 23 Receptor (*IL23R*), endoplasmic reticulum aminopeptidase 1 (*ERAP1*) and RUNX Family Transcription Factor 3 (*RUNX3*) genomic loci elucidating their transcriptional regulation (Keidel et al., 2013; Roberts et al., 2016; Vecellio et al., 2016; Vecellio et al., 2018; Vecellio et al., 2021).

The three-dimensional (3D) organization of the genome is essential in facilitating fundamental processes which occur in the cell nucleus including, transcriptional regulation, DNA damage and replication (McCord et al., 2020). Over the last 20 years, chromosome conformation capture (3C) techniques have been widely used to identify and estimate the frequency of interaction of multiple genomic loci in the genome (Dekker et al., 2002; Phillips-Cremins et al., 2013; Hansen et al., 2018; Haws et al., 2022). In 3C methodology, restriction enzyme digestion followed by re-ligation of cross-linked chromatin in the nucleus of a cell, allows to detect the spatial vicinity between DNA sequences (de Wit and de Laat, 2012). 3C experiments have revealed that chromosomes are folded in complex structures emerging at different scales. These structures can be impacted by disease-associated SNPs, as reported extensively (Gorkin et al., 2019; Anania and Lupianez, 2020; Tsuchiya et al., 2021). Recent studies in polygenic disorders (Girdhar et al., 2022) show that using 3D genome architecture investigation has the utility to clarify the role of disease associated SNPs and to link them to specific genes to understand the phenotype and account for biological function (Khunsriraksakul et al., 2022; Zhao et al., 2022).

Original 3C methods are low-throughput and not able to define if multiple regions interact simultaneously or mutually exclusively. For this reason, several technologies deriving from the standard 3C have been developed, including Hi-C (high-throughput chromosome conformation capture) which allows the analysis of spatial genome organization and chromosome folding through sequencing (Dekker et al., 2002; van Steensel and Dekker, 2010; Dekker et al., 2013).

Here, we explore a selection of 3C methods (i.e. Hi-C, Tiling Capture-C) which might facilitate the understanding of 3D organization and chromosomal interactions and their impact in rheumatic diseases pathophysiology. We emphasise the importance of 3C approaches to inform GWAS interpretation, and their possible future application in precision medicine in prioritizing potential drug targets in polygenic rheumatic disorders including AS.

## The 3D genome and the nuclear architecture

**FIGURE 1:**
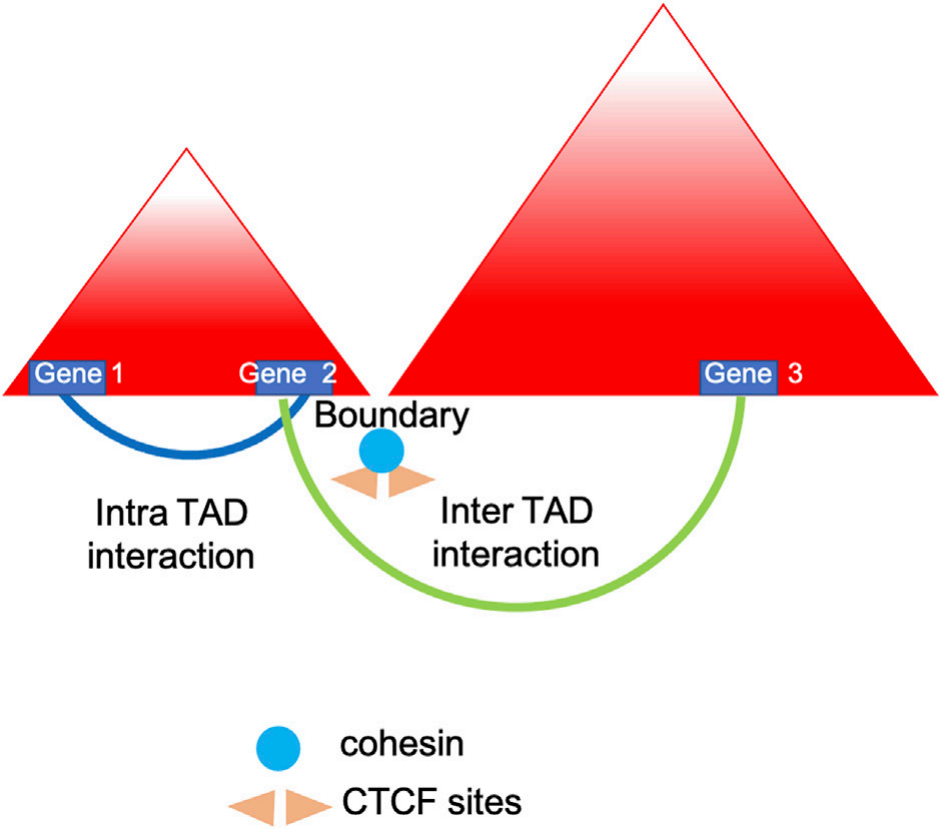
TADs insulation. Simplified model showing how topologically associated domains (TADs) are insulated by borders which are leaky enough to influence and regulate nearby genes expression (Luppino et al., 2020).

Chromosomes fold into compartments, often indicated as A and B, which refer to genomic loci with similar transcriptional activity that physically segregate in 3D space. In addition, contact domains, consist in any visible domains corresponding to elevated chromatin interactions regions. Topologically associated domains (TADs) are the hallmarks of genomic organization (see Figure 1) and are defined as local organizational domains taught to be formed primarily by loop extrusion where boundaries are most conserved during cell differentiation (<1 MB scale) (Gorkin et al., 2019; Goel and Hansen, 2021). TAD dysregulation is linked to various diseases, including neurological disorders and tumorigenesis (Medrano-Fernandez and Barco, 2016; Yang et al., 2019). As demonstrated by Lupianez et al. using CRISPR/Cas9 genome editing and 3C methods, the disruption of TADs might lead to a rewiring of long-range regulatory architectures and result in a pathogenic phenotype. Specifically, the authors focused on rare limb malformations and identified several rearrangements in the *epha4/pax3* (EPH Receptor A4/Paired Box 3) locus in mice causing disruption of the TAD, chromatin structural changes and the aberrant expression of developmental genes (Lupianez et al., 2015; Lupianez et al., 2016). In addition, Luppino and others have demonstrated the loss of cohesin leads to reduced chromatin mixing thus affecting the topology and transcriptional bursting frequencies of boundary-proximal genes (Luppino et al., 2020).

**FIGURE 2:**
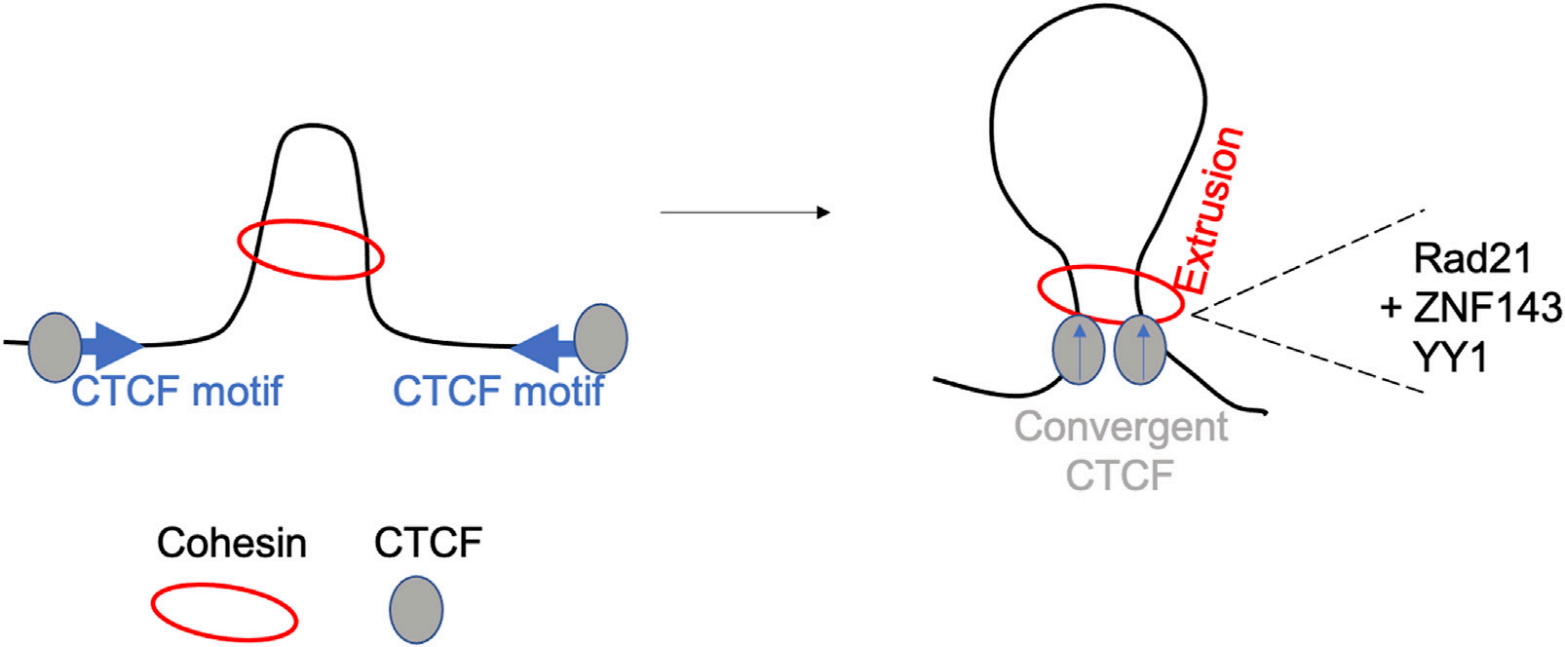
The loop-extrusion model. The model shows the generation of long-range cis-interactions, following extrusion by cohesin, CTCF cognate sites binding and involvement of other accessory proteins including YY1, RAD21 and ZNF143 (Hansen et al., 2018).

Loop boundaries are enriched in CCCTC-binding factor (CTCF) and cohesin, two architectural necessary proteins involved in long-range genome looping. It has been postulated that these two proteins form domains by a loop extrusion process. First proposed in 2015 following mathematical simulations and polymer modelling, the loop extrusion model (see Figure 2) suggests that a chromatin loop of increasing size is extruded by the cohesin complex until it is stalled by a pair of convergently oriented CTCF-bound sites (de Wit et al., 2015; Sanborn et al., 2015). The cohesin complex extrudes a loop uni- or bi-directionally until it faces an occupied CTCF binding site and then the loop is stabilized (Fudenberg et al., 2017). Several studies have explored the interplay between compartmentalization and chromatin looping, showing that the depletion of CTCF has no effect on compartments (Nora et al., 2017), while cohesin (and/or RAD21) removal brings to the loss of domains and TADs making compartmentalization more prominent (Haarhuis et al., 2017; Wutz et al., 2017). In addition to CTCF and cohesin, boundaries frequently colocalize with active transcription start sites along with additional genomic factors such as YY1, RAD21 and ZNF143 which exhibit enrichment at strong boundaries (Hsieh et al., 2015; Bonev et al., 2017). Boundaries which are depleted of CTCF and YY1 are defined as weak boundaries. In addition, chromatin immunoprecipitation (ChIP)-seq experiments have revealed enrichment for ASH2L (ASH2 like, histone lysine methyltransferase complex subunit), H3K4Me3, SP1 (specificity protein 1) among other factors enriched at these boundaries. The role of RNA polymerase II has been investigated by Hsieh and others (Hsieh et al., 2015) demonstrating that active transcription mediated genome folding has a crucial role in the maintenance of the enhancer-promoter and promoter-promoter domains. Following the inhibition of RNA polymerase II, the intensity of those domains is significantly reduced without affecting higher-order chromatin organization (Hsieh et al., 2020). Stable enhancer-promoter interactions have been observed during the formation of *Drosophila* embryos, suggesting these interactions are important in developmental stages, cell fate decision and limb formation (Ghavi-Helm et al., 2014; Ji et al., 2016). Abnormalities in enhancer-promoter interactions, such as mutation in encoding proteins genes or enhancer-binding proteins lead to disease like Cornelia de Lange syndrome, often referred as enhanceropathy (Olley et al., 2018). The process of transcription termination at the 3’ of a gene requires the recruitment of specific factors, which cross-talk with the initiation and enhancement machinery required for the start of transcription. 3C approach has been crucial in demonstrating the formation of gene loops showing interaction between transcription factors associated with promoter and those linked with transcription termination (Al-Husini et al., 2020).

The generation of comprehensive and high-resolution 3D genome maps may facilitate the discovery of small alterations (Chakraborty and Ay, 2019) which can lead a cascade of aberrant molecular events and drive disease phenotype.

## 3C-derived technologies to map the 3D genome

Recent advances in specific methods to investigate the 3D genome architecture include development of high-throughput chromosome conformation capture (Hi-C), Hi-ChIP and Tiled Capture (Mumbach et al., 2016; Galan et al., 2020; Oudelaar, 2022). All three methods capture genomic regions linked *via* 3D interactions, that are detected by Next-Generation Sequencing (NGS).

Hi-C was introduced in 2009, using biotinylation to enrich for proximity ligated contacts and thus modifying the library amplification process. Specifically, HiC takes advantage of using universal adapters and primers for high throughput sequencing. The unbiased “all *versus* all” approach clearly has a real advantage in defining all the genomic interactions genome wide (Lieberman-Aiden et al., 2009). It has been demonstrated that during differentiation, transcriptional changes occur when there is alteration in the strength of long-range interactions and the development of cell-type specific enhancer-promoter contacts (Bonev et al., 2017). Further, these interactions occur primarily in the same TAD and are strongly correlated with gene expression demonstrating how TADs constrain enhancer activity (Symmons et al., 2014).

Recently, Micro-C was developed from Hi-C, moving to a capture-fine (∼1 kb) to a nucleosome level of resolution (∼200bp) (Krietenstein et al., 2020). Formaldehyde and disuccinimidyl glutarate are used for fixation and cross-linking steps, while chromatin is digested with micrococcal nuclease (MNase), instead of restriction enzymes, to reach nucleosome-level resolution. Restriction enzymes sites are not equally distributed and not all the DNA is readily accessible as it is affected by nucleosomal accessibility (Szerlong and Hansen, 2011); using micrococcal nuclease digestion takes advantage of local DNA accessibility and facilitates the retention of intact nucleosomes (Voong et al., 2017). Micro-C has revealed two different classes of CTCF loops: those dependent on RNA-binding region (RBR) and those which are not (Hansen et al., 2019).

Hi-ChIP has been developed to delineate promoter-enhancer interactions by leveraging principles of *in situ* Hi-C (Mumbach et al., 2016), combining long-range contacts investigation with enrichment of specific histone proteins (i.e H3K27Ac) associated with active regions of the genome. Recently, Chandra and others performed Hi-ChIP to provide evidence of non-coding genetic variants having effect on gene expression (i.e. cis-eQTL) and cell-specific gene regulation in five immune cell types (Chandra et al., 2021). Another method coupling ChIP with 3C is ChIA-PET, a chromatin interaction analysis by paired-end tag sequencing (Fullwood et al., 2009). With this approach is possible to detect all chromatin interactions mediated by a specific protein of interest, immunoprecipitated using specific antibodies (Li et al., 2002; Fullwood et al., 2010). In 2013, ChIA-PET was used to described for the first time the genome-wide chromatin interactions of cohesin (DeMare et al., 2013).

Tiling Capture C was designed to identify at high resolution level the interaction between promoter and enhancer within TADs. This method uses a panel of specific capture oligonucleotides tiled across all contiguous restriction fragments within specified genomic regions, typically around 1Mb (Downes et al., 2022), to obtain enrichment for specific interactions and subsequent targeted sequencing. High-resolution maps with low-cells input makes Tiled-C at forefront of the 3C methods. Oudelaar et al. developed the Tiled-C approach to characterize the chromatin architecture of mouse erythroid cells during *in vivo* differentiation, focusing on the *α*-globin locus (Oudelaar et al., 2020). Recently, the same group has generated the most detailed genomic local interaction map at base-pair resolution (20bp), using a micrococcal nuclease (MN) based 3C approach. MN is an enzyme digesting the genome largely independent of DNA sequence: the authors benefit of MN to demonstrate the effects of the depletion of two crucial elements, cohesin and CTCF, on chromatin architecture (Hua et al., 2021; Aljahani et al., 2022). A summary of the different chromatin conformation techniques here presented is showed in Table 1.

**TABLE 1:** A summary of the different techniques routinely used to analyse the conformation of the genome.

*Technique*	3C	4C	Hi-C	Micro-C	Hi-ChIP	ChIA-PET	Tiled-C
*Chromatin looping*	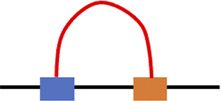	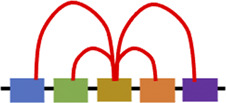	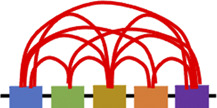	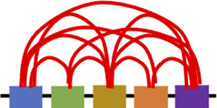	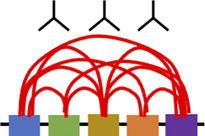	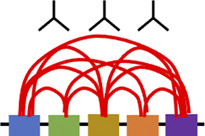	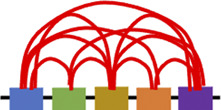
*Type*	One to one	One to all	All to all	All to all	All to all	All to all	All to all
							Some to all
*Main steps*	- Restriction enzyme digestion	- Restriction enzyme digestion	- Restriction enzyme digestion	-Double cross linking	- Restriction enzyme digestion	- Restriction enzyme digestion	- Restriction enzyme or MNase digestion
	- PCR	- microarray	- sonication	- MNase digestion	‐ sonication	‐ sonication	- biotinylated capture oligonucleotides enrichment
			- sequencing	- sequencing	- chromatin IP	- chromatin IP	- sequencing
*Resolution*	standard experiments generate 10–50 kb resolution (locus specific)	from 200–400 bp to 3–4 kb depending on the frequency of four or six base cutter restriction enzymes sites	from 400 bp to 4 kb depending on the frequency of four or six base cutter restriction enzymes sites	∼200bp	From 1kb to 50 kb	from 100 bp to 1 kb	from 20bp to 2 kb (locus specific)
*References*	Dekker et al. (2002)	Meddens et al. (2016)	Lieberman-Aiden et al. (2009)	Szerlong and Hansen, (2011); Voong et al., 2017; Krietenstein et al., 2020	Mumbach et al. (2016)	Fullwood et al. (2010)	Oudelaar et al. (2020)

The investigation of 3D genome topology has dramatically evolved over the past decade: technological advances have boost the field to an unprecedented level (Bouwman et al., 2022). Ongoing efforts are made in providing more accurate data analysis for a better understanding and interpretation of the functional consequence of these changes.

## Investigating the 3D genome in rheumatic diseases and AS

The 3D genome organization has been investigated in autoimmune and rheumatic disease, but more is yet to come. In 2012, 3C was used to identify physical interaction between the chromosome region 16p13, often associated with increased risk for a plethora of autoimmune diseases, including multiple sclerosis (Zuvich et al., 2011), primary biliary cirrhosis (Mells et al., 2011) and systemic lupus erythematosus (SLE) (Gateva et al., 2009), with *DEXI* (dexamethasone-induced protein), a gene with previous unknown function, revealed as a strong candidate for autoimmune disease (Davison et al., 2012).

Meddens and others performed circular chromosome conformation capture (4C)-seq to analyse chromatin interactions in inflammatory bowel disease (IBD) susceptibility loci and DNA regulatory elements providing novel relevant candidate genes (Meddens et al., 2016). In a recent work by Carini and others, the authors explored the genomic architecture of whole blood obtained from rheumatoid arthritis (RA) patients investigating a possible chromosome conformation signature (including *IFNAR1*, *IL-21R*, *IL-23*, *IL-17A* and *CXCL13*), before and after the administration of methotrexate (MTX) treatment. This was important to identify the non-responders to disease modified anti-rheumatic drugs such as MTX and, whether there was an association between the chromatin signature and RA-specific expression quantitative trait loci (eQTL) (Carini et al., 2018). Integrating data from Hi-C with gene expression profiling and disease activity scores has been successful in a recent work on SLE, where the authors established the genomic interaction landscape identifying specific SLE-associated loops (Zhao et al., 2022).

Nearly 90% of disease-associated SNPs are located in non-coding regions (Ricano-Ponce and Wijmenga, 2013). The role of specific GWAS hits can be elucidated *via* 3D genome analysis, thus clarifying which genes are influenced by which particular SNP through a spatial connection (Li et al., 2020). Our group has recently demonstrated the presence of a chromatin loop between the AS-associated SNP *rs4648889* and the distal promoter of the *RUNX3* gene, confirming together with other functional experiments previously reported the primacy of this genetic variant in the association with *RUNX3* in AS (Cohen et al., 2021). The complexity of the *RUNX3* locus is also confirmed by Capture-C experiments showing multiple interactions among different SNPs and the *RUNX3* promoter (personal communication).

In 2015 the Orozco group investigated chromatin interactions between disease-associated genetic variants and their functional targets in B and T cells in four autoimmune disease, including RA, type 1 diabetes, psoriatic arthritis, and juvenile idiopathic arthritis. They performed Capture Hi-C and demonstrated that only few looping interactions were common to both cell lines and disease-associated SNPs interact with candidate genes relevant to the disease and located megabases away (Martin et al., 2015).

It is important to dissect the role of chromatin contacts at diverse genomic loci: the usage of 3D genome structure to perform gene prioritization will be very informative to define new drug targets and evaluate if a therapy is working or a more effective therapy is needed.

As previously demonstrated, genetic variations can influence 3D chromatin conformation, together with accessibility and gene expression (Gorkin et al., 2019). Long-distance eQTLs potentially regulate gene expression and spatial gene regulatory interactions are supposed to be the drivers of the heritability of complex traits. Our completed genome-wide study of chromatin interactions and the regulatory effects of AS-associated genetic variants is unprecedented in the field and it will be very informative in identifying genes and cells to prioritize as therapeutic targets (Brown et al., unpublished data). On the same line, promoter capture Hi-C and RNA-sequencing approaches were recently used to link associated variants of systemic sclerosis (a connective tissue immune-mediated disease) with their target genes, especially in CD4+T cells and CD14^+^ monocytes obtained from 10 patients and five matched healthy controls. The authors identified new potential targets genes and 15 other potential drug targets for repurposing of drugs already in use in other immune-mediated diseases (Gonzalez-Serna et al., 2022).

In RA, a comprehensive genomic map has been recently generated to link risk-associated genetic variants with functional chromatin interactions, active regulatory DNA elements and differential gene expression in fibroblast-like synoviocytes, providing the proof of concept for a causal role of these cells in RA susceptibility (Ge et al., 2021).

The examples provided in this section show once again the importance of a multimodal approach for the identification of cell types and molecular states critically associated with rheumatic diseases. The generation of a comprehensive and high-resolution 3D genome map may yield insights in disease-associated TAD appearance and chromosome loop strengths.

## Discussion

Although chromosome conformation capture is a relatively new field of investigation, understanding 3D genome folding and its influence on gene expression has rapidly grown beside the innovation of 3C methods. The relevance of looping formation and genomic organization together with the identification of architectural proteins associated with boundaries might shed light on the functional and mechanistic implications in different diseases, as demonstrated in these seminal works focused on neurological disorders and cancer (Bharadwaj et al., 2014; Weischenfeldt et al., 2017; Zhu et al., 2020). Further, disease-associated genetic variants may disrupt higher-order genomic organization, due to elimination of annotated boundaries (Ibn-Salem et al., 2014). Normal and disease-associated TAD structure data may yield valuable and perhaps diagnostically important information on gene regulation and disease aetiology. Several diseases could be linked to an aberrant chromatin loop dynamic (Mehrjouy et al., 2018). This suggests an increased interest in studying genome looping and how this may affect gene expression and function in diseases (Krumm and Duan, 2019).

3C approaches might be useful in addressing an unmet clinical need of predicting those patients who will not respond to specific treatments and thus facilitating earlier access to more effective therapies and a better quality of life. The integration of 3C methodologies with functional data (i.e. eQTL) may pinpoint individual loci into a gene regulatory network which is critical to our understanding of complex diseases. Single-cell genomic assays might be a promising tool for the quantification of molecular traits (i.e. transcriptomics, chromatin accessibility, transcription factors occupancy and time-course trajectories of cells) at single cell level (Buenrostro et al., 2015; Zheng et al., 2017; Cuomo et al., 2020; Del Priore et al., 2021). Recently it has been elegantly demonstrated that genetic variants associated with differential binding of PU.1, a master transcription factor regulating myeloid development and having a substantial effect on neutrophils function (McKercher et al., 1996; Siwaponanan et al., 2017), are predominantly cell type specific, associated with specific chromatin state, and regulate enhancer-promoter interactions and downstream gene expression, exhibiting association with IBD susceptibility (Watt et al., 2021). Additionally, PU.1 has been found involved in regulating the interaction loop at *DDX60L* (Probable ATP-dependent RNA helicase DDX60-like) promoter thus inducing overexpression of this gene in CD4^+^ T Cells from SLE patients (Zhao et al., 2022).

The dynamic interplay between genomic sequence, 3D chromatin structure and a specific pathological process such AS, is crucial in defining a powerful strategy to discover novel genetic regions potential for diagnostic or therapeutic purposes. Nevertheless, it is important to bear in mind that a combination of more genetic variants (i.e. haplotypes) may have a higher risk on the probability of developing a particular disorder, while individually may have a mild influence.

## Concluding remarks

The last decade has been a remarkable time for genetic research in AS, from genetic association studies to genetics-driven novel clinical trials (Baeten et al., 2013; van der Heijde et al., 2019; Izana Bioscience, 2021). The identification of few hundreds genomic loci make the overall analysis challenging, considering most of them are enriched not only in immune cell-specific enhancers, but also in osteoclasts or stromal cells, having a crucial involvement in the pathogenesis of AS. Contemplating the results of GWAS suggests that a traditional *in vitro* approach to uncover the mechanistic contribution of disease-associated SNPs is not sufficient anymore. Times for integration of genetic fine mapping of AS loci with DNA architecture and 3D chromatin interactions, DNA accessibility, single-cell gene expression and gene editing are mature as they occur in related disorders (Al-Mossawi and Coates, 2018; Penkava et al., 2020; Yager et al., 2021), and it appears only a matter of time before they are fully applied in AS.

## Data Availability

The original contributions presented in the study are included in the article/supplementary material, further inquiries can be directed to the corresponding author.
